# Comparison of IPV to tOPV week 39 boost of primary OPV vaccination in Indian infants: an open labelled randomized controlled trial

**DOI:** 10.1016/j.heliyon.2016.e00223

**Published:** 2017-01-09

**Authors:** Suman Kanungo, Deok Ryun Kim, Bisakha Haldar, Cynthia Snider, Uma Nalavade, Soon Ae Kim, Ju Yeon Park, Anuradha Sinha, Aiyel Haque Mallick, Byomkesh Manna, Dipika Sur, Ranjan Kumar Nandy, Jagadish M. Deshpande, Cecil Czerkinsky, Thomas F Wierzba, William A Petri Jr., Mohammad Ali, Ayan Dey

**Affiliations:** a National Institute of Cholera and Enteric Diseases, Kolkata, India; b International Vaccine Institute, Seoul, South Korea; c Cone Health, Greensboro, NC, USA; d Enterovirus Research Centre, Mumbai, India; e Institut de Pharmacologie Moleculaire & Cellulaire, CNRS-INSERM-University of Nice-Sophia Antipolis, Valbonne, France; f PATH, Washington, DC, USA; g Division of Infectious Diseases and International Health, The University of Virginia, Charlottesville, VA, USA; h Johns Hopkins Bloomberg School of Public Health, Baltimore, USA

**Keywords:** Health profession, Immunology, Pharmaceutical science, Pediatrics, Public health, Internal medicine, Pathology, Infectious disease

## Abstract

**Background:**

The final endgame strategy of global polio eradication initiative includes switching from trivalent oral poliovirus vaccines (tOPV) to bivalent oral polio vaccine (bOPV), and introduction of inactivated poliovirus vaccine (IPV). This study compares IPV with tOPV week 39 boost in Indian infants.

**Methods:**

Starting 28 March 2012, we enrolled 372 Indian infant-mother pairs from Kolkata, India in an open-label, block-randomized, controlled trial comparing a 39 week tOPV to an IPV boost among infants immunized with three doses of tOPV. The primary outcome was mucosal immunity to poliovirus as measured by fecal polio virus shedding after OPV challenge. The secondary outcome was humoral response as defined by >1:8 titers for neutralizing antibodies at week 40. Seroconversion was measured by change in level of antibody titers from week 18 to week 40. The analyses were performed by both intention-to-treat (ITT) and per-protocol (PP) comparing the occurrences of outcomes between the arms of the study.

**Findings:**

Both the study arms provided equivalent mucosal immunity at 52 weeks with a total shedding prevalence of 28%. Vaccination with IPV resulted in significantly higher seroconversion rates for Polio type 2 (p = 0.03) and Polio type 3 (p < 0.01).

**Conclusions:**

This study indicates that an IPV boost at week 39 is equivalent to tOPV in intestinal immunity, and provides higher seroconversion compared to tOPV. The major limitation of the study was the additional OPV doses receive by infants during pulse polio immunization resulted in additional mucosal boosting, diminishing the impact of IPV or tOPV boost at week 39. However, IPV for OPV boost should prove to be a step forward in the global polio eradication initiative to reduce the problem of circulating vaccine-derived poliovirus (cVDPV).

## 1 Introduction

The global eradication of poliomyelitis is close at hand. The year 2015 marked the lowest incidence of paralytic polio since eradication effort were initiated. Only two countries, Afghanistan and Pakistan reported wild poliovirus disease [1]. In 2015, 74 wild poliovirus (WPV) cases were identified; 54 (73%) were detected in Pakistan, and 20 (27%) were detected in Afghanistan. In 2016 unfortunately three wild poliovirus type 1 (WPV1) cases have been reported from Borno State of Nigeria [2]. In addition to the WPV, about 32 circulating vaccine-derived poliovirus (cVDPV) cases were reported from polio-free countries. Outbreaks of cVDPV type 1 occurred in Laos, Madagascar, and Ukraine, whereas outbreaks of cVDPV type 2 reported from Guinea, Myanmar, Nigeria, and Pakistan [1].

One of the reasons for continued transmission in these 3 countries may be the lower immunity generated by oral polio vaccine (OPV) in children from resource poor settings [3, 4]. This poor protection following OPV vaccination is thought to be multi-factorial with contributions from chronic diarrhea from concurrent enteric infections and potentially maternal antibody interference with vaccine antigen uptake [5, 6, 7].

The polio eradication and endgame strategic plan of 2013–2018 had reached milestones with the second objective that includes switching from the trivalent OPV (tOPV) to bilavent OPV (bOPV, composed of serotype 1 and 3) to target the remaining circulating wild polio virus serotypes [1]. In 2016, 154 countries decided to switch from tOPV to bOPV in their routine and supplementary immunizations [8]. In addition to switching from tOPV to bOPV, introduction of one dose of inactivated polio vaccine (IPV) into the routine immunization programs has been recommended for maintaining type 2 poliovirus immunity [9].

IPV has been used successfully in many polio-free countries to maintain humoral immunity in children [10]. IPV has also been used in supplementary immunization activity (SIA) to control outbreaks and accelerate poliovirus eradication in endemic countries [11, 12]. Vaccination with only IPV was apparently inferior as measured by fecal shedding after challenge, but reportedly had different outcome when administered subsequent to doses of OPV [13, 14, 15]. Although many of these studies support the use of IPV after OPV, the timing of IPV and the effect of it on mucosal protection represented a knowledge gap.

We designed a randomized clinical trial to determine if an additional dose of IPV or tOPV administered at 39 weeks of infant age after receiving 3 doses of tOPV, would boost mucosal and humoral immune responses, as measured by fecal viral shedding for all the three poliovirus type at week 52 (day 0 to day 25) after OPV challenge, and by neutralizing antibodies at week 40. We hypothesized that infants receiving the IPV dose after OPV intestinal priming would enhance intestinal and humoral immunity with reduced viral shedding of poliovirus.

## 2 Materials and methods

### 2.1 Study population and design

The study was approved by the Indian Council of Medical Research (ICMR), Health Ministry's Screening Committee (HMSC), Government of India; the Institutional Ethical Committee at National Institute of Cholera and Enteric Disease (NICED) and the Institutional Review Boards of International Vaccine Institute (IVI), South Korea; University of Virginia and University of Vermont USA. The study protocol was registered at clinical trial registry of India (CTRI/2012/03/002504) and at clinicaltrials.gov (ClinicalTrials.gov Identifier: NCT01571505). All participants received free primary care during the study. All mothers signed an informed consent form on behalf of their infants before enrollment into the study and were free to withdraw at any time.

Three-hundred seventy-two eligible neonatal infant-mother pairs were enrolled into the Performance of Rotavirus and Oral Poliovirus Vaccines in Developing Countries (PROVIDE) study in Kolkata, India. It was a randomized open-label clinical trial. Recruitment of the subjects was over one and half year starting on 28th March 2012 and ending in 30th October 2013. The subjects were followed-up for a period of 53–54 weeks of infant age with the final follow-up visit completed on 30th November 2014.

**Fig. 1:**
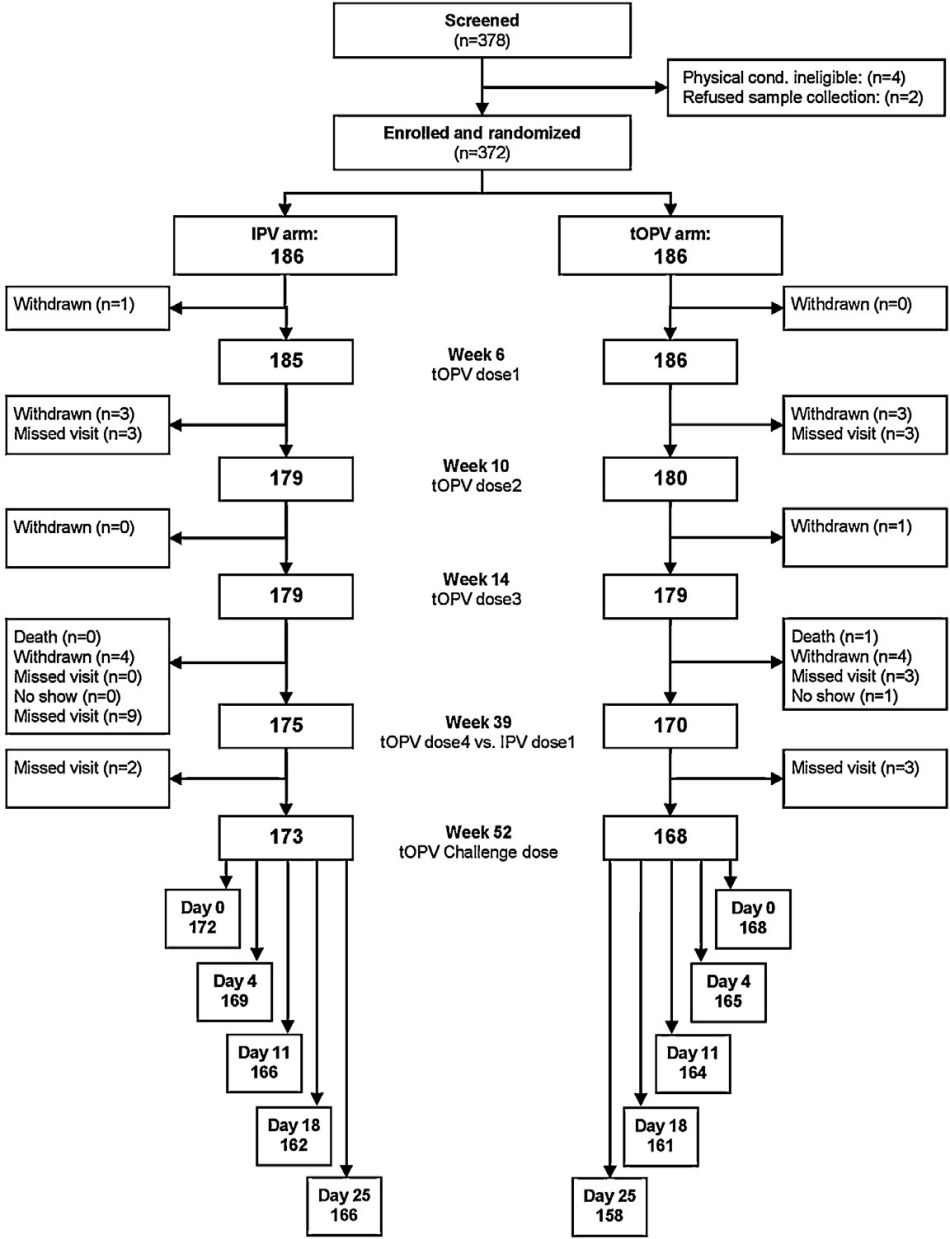
Consort Diagram for the PROVIDE study, Kolkata, India.

The study design was a randomized open-label clinical trial with 2 arms. Infants enrolled received three doses of tOPV followed either by a fourth dose of tOPV or IPV followed by a challenge dose of tOPV (Fig. 1). All participating infants received OPV and BCG at birth, and diphtheria, pertussis, tetanus (DPT) vaccine, Hepatitis B vaccine and OPV at 6, 10 and 14 weeks of age as part of expanded program of immunization (EPI). The study groups were: i) dose 4 IPV plus Rotavirus and ii) dose 4 tOPV plus Rotavirus. Subjects were randomized to receive either oral tOPV (BioPolio^®^, Bharat Biotech) or injectable IPV (IMOVAX^®^, Sanofi Pasteur) at 39 weeks of age. The reason for selection of 39 week boost was to harmonize with PROVIDE study arm of Bangladesh, where the EPI schedule for oral poliovirus vaccination (tOPV) at the time of the study design in 2010 was 6, 10, 14, and 38 weeks [16, 17]. The fourth tOPV dose was administered at 39 weeks in this trial but was within the preferred vaccination window [16, 17]. Another challenge dose of tOPV were given to all infants at week 52 age (Fig. 1). Additionally, all subjects were vaccinated with Rotarix^®^ (GlaxoSmithKline) as the part of the study (Supplementary Table 1). Co administration of polio and rotavirus vaccine were assumed to have no effect on the poliovirus outcomes [18].

Mothers with newborns and who visited the B. C. Roy Children’s Hospital, Kolkata, India for routine expanded program of immunization (EPI) were screened for eligibility and enrolled by the project staff. Eligibility for enrollment required infants aged 0–49 days, absence of congenital abnormalities, mother and child not immunologically compromised, no birth defects, no history of seizures and other apparent neurologic disorders, and proof of BCG and OPV vaccination in their immunization cards. All mothers signed an informed consent form on behalf of their infants before enrollment into the study and were free to withdraw anytime at their will. The study was conducted in compliance with the declaration of Helsinki and the Belmont Report [19]. Good Clinical Practices were followed throughout the progress of the study. Adverse events were monitored by an independent clinician who was not involved in the study. Severe adverse events were recorded for the full protocol period until the final fecal excretion sample collection at 53–54 weeks of age; and adverse events within 48 h of vaccination linked to vaccine administration were recorded. All adverse events were reported to ethics review committee and institutional review board as per protocol.

### 2.2 Randomization and masking

Infants were randomized to receive either tOPV or IPV at 39 weeks of age. All infants received tOPV at birth, 6, 10 and 14 weeks of age. Based on the randomization codes generated at the International Vaccine Institute (IVI), designated staff at National Institute of Cholera and Enteric Diseases (NICED) had assembled and numbered envelopes with group assignment (i.e. Group A or Group B). These were sealed and signed by the study investigator, delivered to the PROVIDE study clinic at B C. Roy Hospital. We had prepared a randomization list with the envelopes containing randomization numbers, Group Code, Study ID (SID) number, and the date of recruitment. The randomization numbers began at “RN1001” and end at “RN1372”. SIDs were assigned sequentially to each infant/mother pair at the enrollment visit. The envelope was opened at the infant’s week 6 visit. Neither mothers nor clinic staff were masked. However, the laboratories that performed detection of fecal poliovirus in stool samples by cell culture and serum neutralizing antibody (Enterovirus Research Centre) were masked or blinded to trial arm assignment.

### 2.3 Study procedures

The poliovirus vaccine cell culture-based fecal excretion assays were performed at the Enterovirus Research Centre (EVRC), Government of India, Mumbai as per the WHO Polio approved assay procedure [20]. Confirmation and strain identification were done by RT-qPCR on identified typed polioviruses of high titre in cell culture [20, 21]. Only the poliovirus samples confirmed positive in cell culture were tested for virus identification using PCR. Samples were also tested for serum neutralizing antibodies (SNAb) at the Enterovirus Research Centre (Mumbai, India) using a standard micro-neutralization assay for antibodies to poliovirus types 1, 2, and 3 according to established protocols [22]. HEp-2 cells were inoculated and each specimen was run in triplicate in the same assay run and results were recorded. A titer of 1:8 or greater was considered seropositive while a titer of less than 1:8 was considered seronegative. As per WHO definition seroconversion was defined as a change from undetectable to detectable titer; or a four-fold increase in titer over the expected decrease in maternally derived antibodies (assuming a half-life of 28 days) [9]. We had also tested the SNAb week 40 measures of seropositivity as predictive correlates of future week 52 shedding.

### 2.4 Outcomes

The primary outcome was the fecal excretion of the any of the three Sabin poliovirus vaccine types determined by cell culture in any of five fecal samples collected at day 0 (immediately before vaccination), 4, 11, 18, and 25 after a 52 week tOPV challenge. We confirmed the polio excretion outcomes through direct fecal RT-qPCR detection. Further RT-qPCR-based quantitation was done as total viral shedding by poliovirus type. The secondary outcome was the humoral immune response for neutralizing antibody to each serotype at one-week post-intervention dose (40 weeks of age) and seroconversion from 18 to 40 weeks.

### 2.5 Statistical analysis

For sample size calculations, we assumed that the tOPV arm had a higher fecal poliovirus-shedding rate at week 52 than IPV. Using a two-sided test at alpha = 0.05, 80% power, 15–20% shedding in tOPV arm and 5% shedding in IPV arm [23, 24] and a 30% loss to follow-up, we required a sample size of 372 infants (186 per arm) [25].

The seropositive humoral response was defined as > = 1:8 titers for neutralizing antibody at week 40. The seroconversion from week 18 to week 40 was defined as a + 2 increase in log titers based on the level of antibody titers at week 18. The week 18 SNAb titer was adjusted for residual maternal antibody levels, assuming 28 days half-life for maternally transmitted antibody measured at 6 weeks of age [16]. Therefore, the adjusted week 18 titer (a18) was calculated as a18 = (s18–s6) + d/28, where, s18 is Log_2_ of titer at week 18, s6 is Log_2_ of titer at week 6, d is number of days between week 6 and week 18 visit. The non-seroconversion was defined as less than 2 change in week 40 titer if:

i. log_2_ SNAb (week 40) ≤2.83; if adjusted SNAb (week 18) ≤2.83;
ii. log_2_ of SNAb (week 40) minus log_2_ of SNAb (week 18)] <2; if adjusted SNAb (week 18)>2.83 and ≤8.5;
iii. log_2_ SNAb (week 40) <10.5; if adjusted SNAb (week 18)>8.5 and <10.5; [16, 26].

Infants with log_2_ titer 10.5 at week 18 were excluded from seroconversion analyses. Detection by this SNAb ranges from 2.5 log_2_, or “negative,” to 10.5 log_2_, as highest tested value [27].

The weight for height (WHZ) was measured using the World Health Organization (WHO) Child Growth Standards. Moderate wasting was defined as a WHZ between -3 and -2 SD, and the severe wasting was defined as a WHZ of below -3SD [28].

The primary analysis was performed intention-to-treat (ITT) comparing the occurrences of outcomes between the arms using tests of proportion. We applied Wilson method [29] for one proportion and Newcombe hybrid score for two proportions [30]. We had also performed per-protocol (PP) and received protocol dose (RPD) analyses for both primary and secondary outcomes to assess possible retention bias. The PP analysis included recipients of correct number doses and in right time as described in the protocol. The RPD analysis included recipients of correct number of doses but one or more visits outside visit windows (Table S3). In the ITT analysis, we had assumed missing samples would have tested negative, resulting in an under-estimation of true poliovirus shedding.

To test for difference in proportions such as dichotomous variables of baseline characteristics, number of shed and incidences of adverse events between the two arms or between seropositive and seronegative SNAb group were evaluated using Chi-Square and Fisher’s exact test. The homogeneity of frequency of additional OPV doses between arms was tested using Chi-Square test and the mean number of additional OPV doses between arms was tested using T-test. All statistical analyses were performed SAS 9.4 (SAS Institute, Cary NC) and the forest plots of risk difference were drawn using R version 3.2 [31].

## 3 Results

Study participants were recruited from clinic where previous study trials were conducted and had high rates of participation. Between March 28th, 2012 to October 30th, 2013, 378 pairs of mother and infant were screened for eligibility. Of those, 372 mother-infant pairs were randomized to each trial arm, comprised of 186 pairs. At enrollment the infants mean age were 44 days (range 42–49 days), 90% were born at a hospital. 96.0% of the families had a toilet or septic tank and 100% had access to municipality water supply (Table S2). The subjects were from better socio economic background as evidence from the average family expenditure of approximately INR 6000/-, parents education and having luxury items at home. All this gave them high score as per earlier publication of socio economic status in India [32]. However, only 11.0–14.0% percentage of the household used treated water before drinking (water filter, solar disinfection, boil, strain through cloth, add bleach/Chlorine). 39.0–41.0% of the subjects had open drain besides their home (Table S2). Over the study period of 6 to 54 weeks, 5–10% of infants were moderately-to-severely undernourished as defined by WHZ< -2(mean WHO WHZ <-2). The week 52 tOPV challenge dose was given to 341 infants (91.7%) with 173 subjects in IPV arm and 168 in tOPV arm (Fig. 1). Nearly seventy percent (259/372 infants) met the PP criteria; while ninety one percent (340/372) fall into the RPD group (Table S3).

There were 30 SAEs events including one death during the study up to the last day 25 fecal sampling visit after week 52 (Table S4). These SAEs were caused by diarrhea and respiratory illness. One death reported was a cot death of unknown etiology. Verbal autopsy were done and reported to IRB for the death. None of the cases were related to vaccination as assessed by the independent medical monitor. Expected mortality and morbidity rates were observed in this population. There were no differences in the SAE counts between the arms (11/186 in IPV and 9/186 in OPV, p = 0.65), as well as in the vaccination-related adverse event (AE) counts (3/186 vs 1/186, p = 0.62).

The number of additional OPV doses during Pulse Polio immunization program [33] until 40 weeks of age was compared between the arms. Nearly all 98% infants had received at least one additional OPV dose and 43% infants received four additional doses. The number of additional doses and the mean doses were not significantly different (p > 0.5) between the arms (Table S5).

In ITT analysis, comparison of IPV boosting to tOPV at 39 weeks of age did not result in any difference in shedding for any of the poliovirus serotypes as measured from 5 fecal samples taken at different days after week 52 tOPV challenge (Table 1). The risk difference was 0.0% between two arms with an inferred equivalence margin of −8.0%, +7.6% (95% CI). Total fecal shedding rate was 28% for both the arms.

**Table 1:** Prevalence of fecal excretions by trial arm (Intention–to- treat analysis).

	No. shed/total (%)		Risk Difference	Relative Risk	P value
	IPV arm	tOPV arm	(95% CI)	(95% CI)	
Cell culture	52/186 (28%)	52/186 (28%)	0.0% (-8, 7.6)	1.00 (0.72, 1.39)	1.00
Cell culture (infants with complete shedding data)	49/152 (32.2%)	45/147 (30.6%)	1.6% (-7, 10)	1.05 (0.75, 1.47)	0.76

Note: The endpoint is the presence of any poliovirus Sabin type in any of the 5 fecal samples taken at 52 weeks of age, measured as the prevalence among infants in each arm (Prev%). Results are shown for the presence of poliovirus detected by cell line culture.

**Fig. 2:**
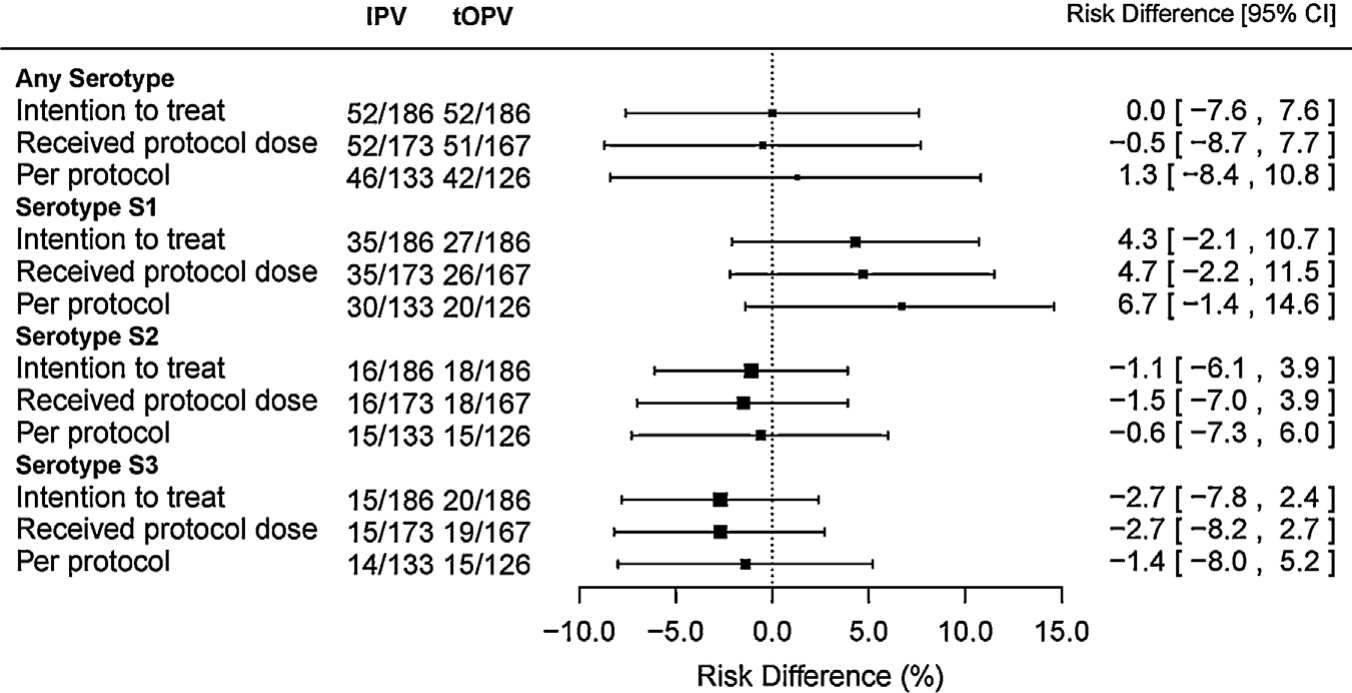
Poliovirus fecal excretion results at 52 weeks of age by type of analysis. Note: Numerators were the number of infants excreting the poliovirus serotype at any 5 time points of the post-tOPV challenge. A serotype refers to shedding of any of the 3 serotypes. Points are the estimated risk difference and the size of a point accounts for the precision of the estimates. Two-sided bars delineate 95% CIs. Risk differences are shown as percentages. Negative (−) values imply lower shedding in the IPV arm compared to that in the tOPV arm.

**Fig. 3:**
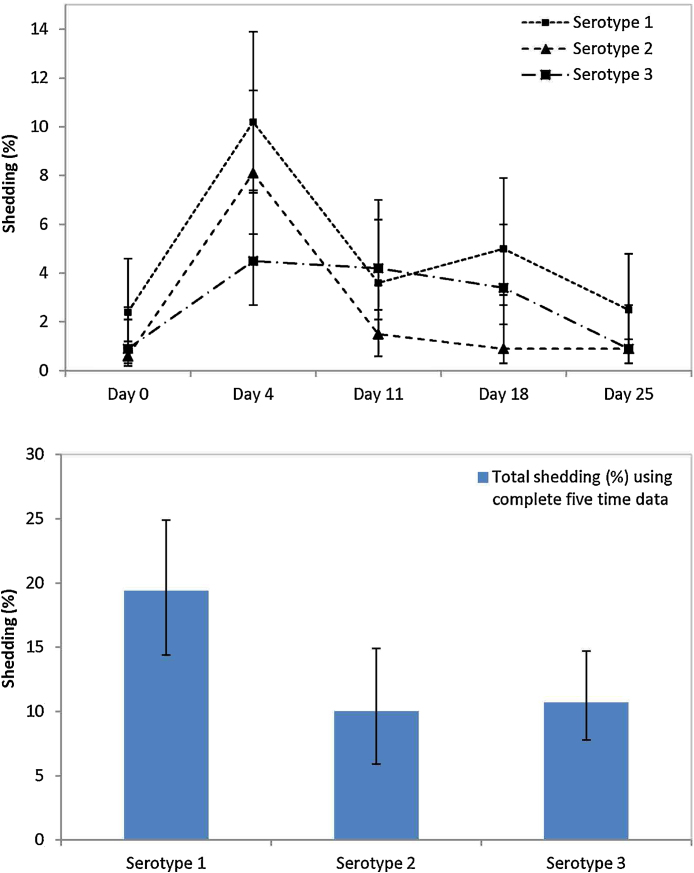
Prevalence of poliovirus fecal excretion by time point and serotype. Note: Results are shown for the combined data and for all infants in both trail arms. The prevalence of shedding at each time point was estimated using all available data points. The total prevalence of shedding by serotype was estimated using only infants with complete five time data.

Secondary analysis of individual serotype fecal shedding were done for PP and RPD to identify differences between IPV and tOPV (Fig. 2). The results of the PP and RPD analyses for shedding by serotype were also consistent with the ITT analysis (Table 1 and Table S6). Estimated total shedding was 31.4% with risk difference of 1.6% (95% CI: −7% to 12%). Repeating the same analyses using fecal RT-PCR-based detection yielded identical results. (Table 1). Infants poliovirus fecal shedding were predominant on day 4, 11 and 18 (Fig. 3). The peak shedding rate was 19% on day 4, 8% on days 11 and 18 for all three poliovirus types (Table 2).

**Table 2:** Poliovirus fecal excretion by time point and serotype.

Serotype	No. shed/total (%)					
	Day 0	Day 4	Day 11	Day 18	Day 25	Total
Any	12/340 (3.5%)	64/334 (19.2%)	27/330 (8.2%)	27/323 (8.4%)	13/323 (4%)	94/299 (31.4%)
1	8/340 (2.4%)	34/334 (10.2%)	12/330 (3.6%)	16/323 (5%)	8/323 (2.5%)	58/299 (19.4%)
2	2/340 (0.6%)	27/334 (8.1%)	5/330 (1.5%)	3/323 (0.9%)	3/323 (0.9%)	30/299 (10%)
3	3/340 (0.9%)	15/334 (4.5%)	14/330 (4.2%)	11/323 (3.4%)	3/323 (0.9%)	32/299 (10.9%)

Note: Prevalence of poliovirus fecal excretion by time point and serotype. Results are shown for the combined data of all infants in both arms. The prevalence of shedding at each time point was estimated using all available data points. Total prevalence of shedding by serotype was estimated only for infants with all five time points.

**Fig. 4:**
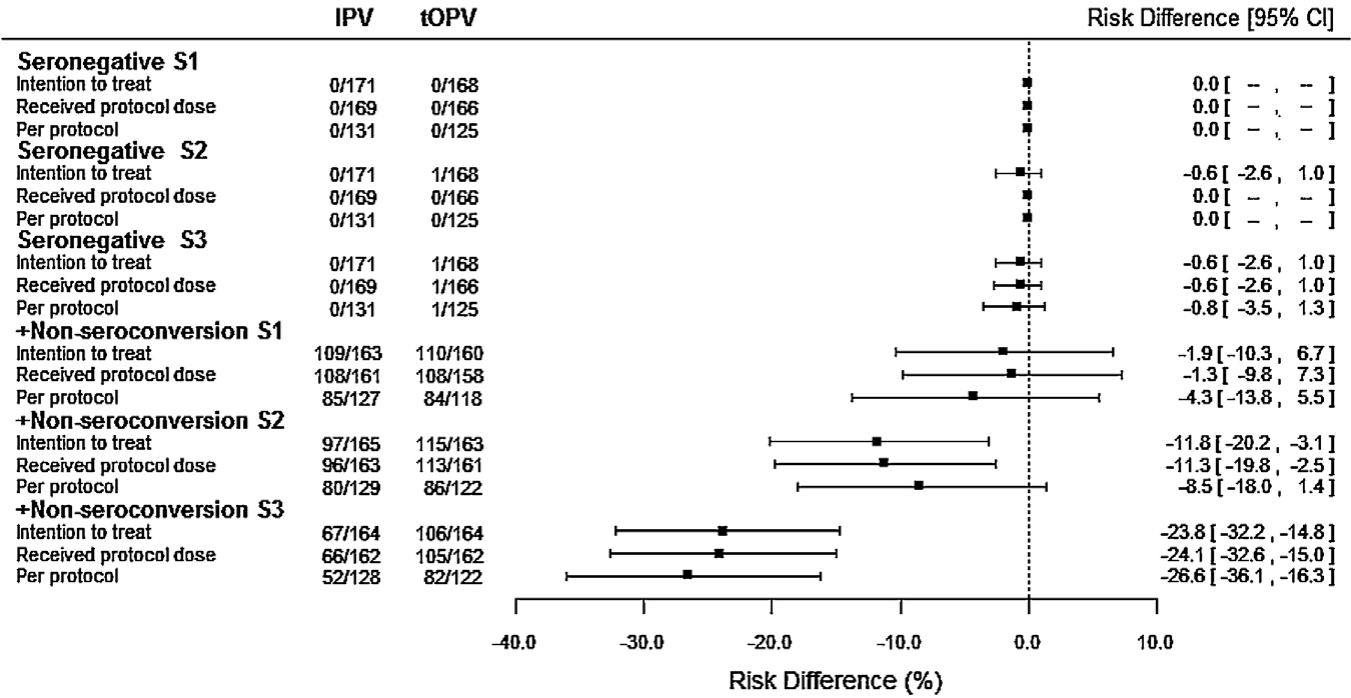
Serum neutralizing antibody (SNAb) for IPV and tOPV by analysis type. Note: SNAb, a secondary outcome, measured in serum from age week 40 infants after receiving IPV and tOPV doses at week 39. The outcomes were defined to focus on possible vaccine failure. Seronegative infants had no detectable SNAb at week 40. Seroconversion failure are infants who did not seroconvert between week 18 (post tOPV dose 3) and week 40, adjusted for residual maternal antibody.

**Fig. 5:**
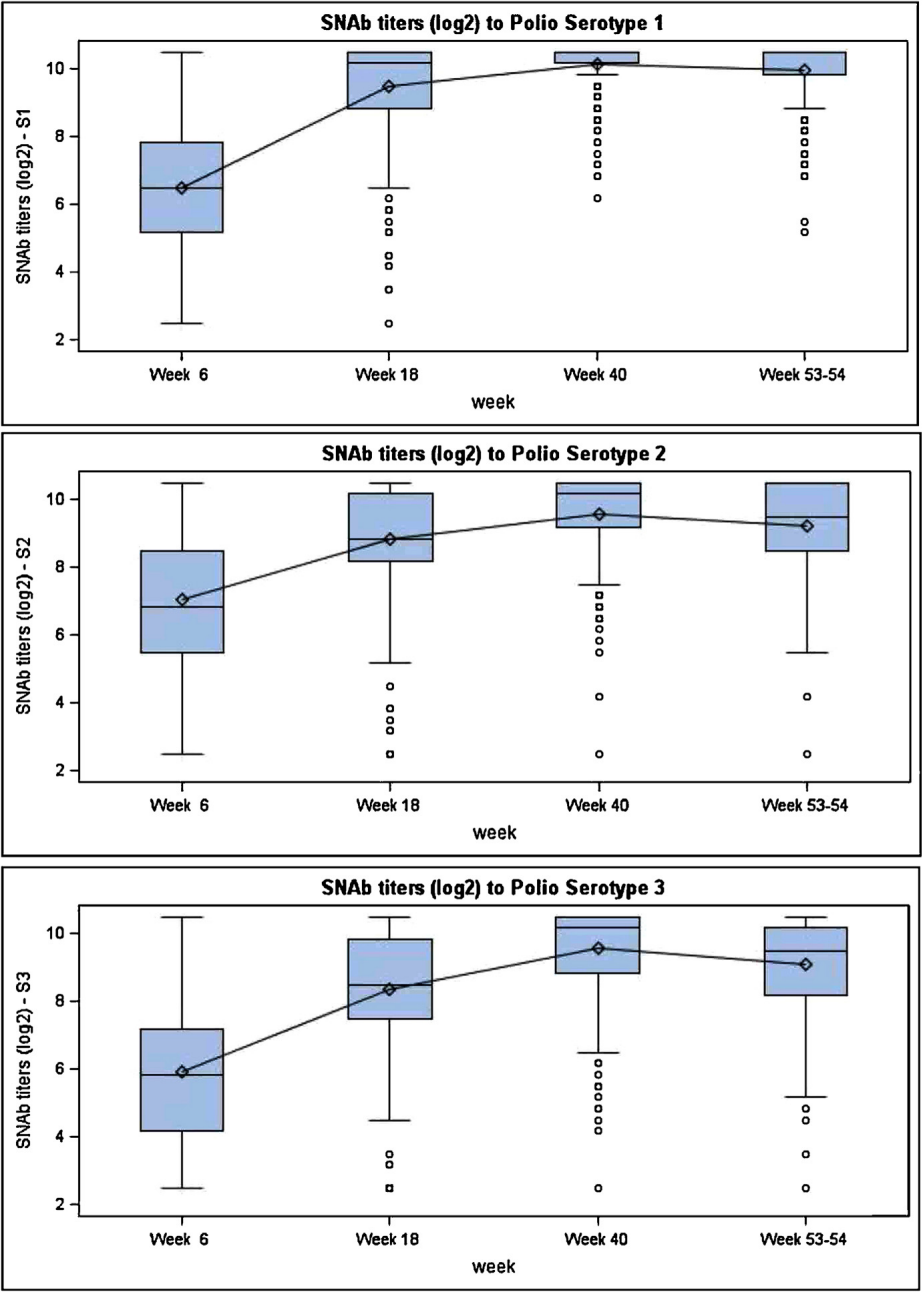
Boxplot of polio neutralizing antibody titers (log2) for all the subjects during Week 6 and Week 53–54 of infants’ age.

In ITT analysis, vaccination with IPV had resulted in no different seronegativity rates for all three serotypes compared to tOPV (Fig. 4). Seronegativity rates in both arms were 0% for type 1 and 0% (IPV) vs 0.6% (tOPV) for type 3 (p = 0.496). No difference in Type 2 seronegativity was observed with IPV and OPV seronegativity of 0% and 0.6% respectively for each arm (p = 0.496). Moderate seroconversion rate from week 18 to week 40 was observed for infants in the IPV arm with a 34.0–60.0% seroconversion of all three antibody types. However for the tOPV arm the seroconversion rate ranged from 30.0–35.0% for all the three serotypes (Fig. 4). Seroconversion rates in IPV compared to tOPV arm were significantly higher for polio type 2 (p = 0.03) and polio type 3 (p < 0.01). For polio type 1 type the seroconversion rates were not significantly different for IPV and tOPV arm (p = 0.72). High serum neutralizing antibody titers were observed over period from week 18 to week 53–54 (Fig. 5). The analysis of week 40 SNAB titers, as predictor of week 52 shedding, showed that infants with seroconversion at week 40 from week 18 were similarly shed at week 52 than those who were not seroconverted at week 40 (Table 3). The shedding difference between those who were seroconverted and those who were not seroconverted was not statistically significant. Seropositivity for each polio type among those infants who shed at week 52 were similar between IPV and tOPV arm (Table 3).

**Table 3:** Seropositivity and seroconversion by virus type measured at age of week 40 as correlate of poliovirus shedding at age of week 52.

Study arm	Week 40 serum neutralizing antibody measure*	Serotype	No. shed/total (%) with +ve SNAb	No. shed/total (%) with -ve SNAb	p-value
IPV	Seropositivity	1	33/150 (22%)	0/0 (N/A)	N/A
		2	15/150 (10%)	0/0 (N/A)	N/A
		3	14/150 (9%)	0/0 (N/A)	N/A
	Seroconversion	1	11/46 (24%)	21/96 (22%)	0.831
		2	8/60 (13%)	7/84 (8%)	0.410
		3	7/83 (8%)	7/60 (12%)	0.576
tOPV	Seropositivity	1	24/146 (16%)	0/0 (N/A)	N/A
		2	15/146 (10%)	0/0 (N/A)	N/A
		3	18/145 (12%)	0/1 (0%)	<0.001
	Seroconversion	1	7/42 (17%)	15/98 (15%)	0.806
		2	4/43 (9%)	11/100 (11%)	1.000
		3	7/51 (14%)	11/92 (12%)	0.796

## 4 Discussion

In this study we observed that substitution of IPV for tOPV with the 39 week booster resulted in improved serum neutralizing antibody without any change fecal excretion of sabin poliovirus strains (upon subsequent challenge with tOPV at 52 weeks). We concluded that introduction of an IPV boost into the EPI program in India would be equally beneficial to the existing OPV boost. The results of the PP and RPD analyses were also consistent with the ITT analysis suggesting the results of our analysis were robust.

Our study extended previous works which tested combinations of OPV and IPV. An IPV dose had been shown to boost seropositivity and seroconversion in children and infants receiving multiple OPV doses [9, 16, 24, 34, 35]. Moreover, the combination of IPV and OPV provided comparable intestinal immunity to OPV, but better than IPV alone [15, 24]. A recent study carried out in western and southern part of India demonstrated that the combination of OPV and IPV had superior immunogenicity for poliovirus type 3 over tOPV only [9]. This supports our finding of a significantly higher seroconversion rate for polio type 3 with the IPV boost. Another study carried out in the Moradabad district of northern India demonstrated that administering a single dose of IPV in OPV vaccinated children improved both humoral and intestinal immunity and helped to overcome waning of mucosal immunity in these children [34].

Jafari and his colleague demonstrated IPV to be superior to bOPV in reducing any shedding in three cohorts of children aged 6–11 months, 5 and 10 years [34]. Unlike our results, a significant reduction in polio type 1 and 3 excretion was observed in the 6–11 month cohort of IPV recipients compared to non-vaccinated controls or bOPV recipients [34]. One of the reasons could be geographic variations of immunogenicity to OPV. Further, all the subjects in our study received additional OPV doses during Pulse Polio immunization program [33] along with EPI vaccines resulting in high antibody titers and comparable excretion rate in tOPV and IPV arm. Moreover, an additional birth dose of OPV might have resulted in high SNAB antibody titers at week 18 and maintained it over time with additional OPV doses.

Our findings are consistent with the results of the larger site of the PROVIDE study in Dhaka, Bangladesh [16, 17], which showed that the proportion of subjects who seroconverted were significantly higher in tOPV plus IPV group compared to only tOPV group while no difference in fecal excretion rates was observed.

Several potential limitations in our trial should be acknowledged. First, the recruitments were conducted in a hospital setting where mothers were, as shown by the high rate of participation, willing to participate. Moreover, the participants were with a better nutritional status and only 5–10% of infants were moderately-to-severely undernourished as mentioned above. This could be a selection bias and limit the generalizability as there are reports of 45% undernutrition from different localities in Kolkata [36]. Second, about 15–20% of the participants in each arm of the study did not provide all stool specimens as outlined in the study protocol. However, the primary ITT and secondary PP and RPD analyses did not showed any significant difference suggesting no effect of the missing stool samples in our study. Third all the participants received additional OPV doses during the Pulse Polio immunization program [33], although the mean number of doses received per subject was constant between the arms. This additional OPV doses may have resulted in additional mucosal boosting, diminishing the impact of additional IPV or tOPV boost. Fourth, this study was limited to measuring immune response only at 12 weeks after IPV vaccination, thus potential long-term changes or waning immunity over time were not addressed.

India EPI has introduced one dose of IPV at week 14 age in 17 high-risk states and four Union Territories from November 2015 and announced tOPV-bOPV switch from mid-2016. This study is timely in assessing the effects of IPV introduction on poliovirus shedding and subsequent intestinal immunity. Though IPV does not appear to be a superior regimen over OPV, the IPV switch is expected to prevent the poliomyelitis caused by cVDPV2, which contributes to 95% cVDPV worldwide. Further studies are warranted to measure the duration of memory response for the IPV plus OPV regimen.

## Declarations

### Author contribution statement

Suman Kanungo: Conceived and designed the experiments; Performed the experiments; Analyzed and interpreted the data.

Deok Ryun Kim, Mohammad Ali, Ayan Dey: Conceived and designed the experiments; Analyzed and interpreted the data; Wrote the paper.

Bisakha Haldar: Performed the experiments.

Cynthia Snider: Wrote the paper.

Uma Nalavade, Jagadish M. Deshpande: Performed the experiments; Analyzed and interpreted the data.

Soon Ae Kim: Conceived and designed the experiments; Performed the experiments.

Ju Yeon Park: Analyzed and interpreted the data.

Anuradha Sinha, Aiyel Haque Mallick: Contributed reagents, materials, analysis tools or data.

Byomkesh Manna: Analyzed and interpreted the data; Contributed reagents, materials, analysis tools or data.

Dipika Sur, Cecil Czerkinsky, Thomas F. Wierzba, William A. Petri Jr.: Conceived and designed the experiments.

Ranjan Kumar Nandy: Conceived and designed the experiments; Contributed reagents, materials, analysis tools or data.

### Funding statement

This work was supported by University of Virginia, through grant from Bill and Melinda Gates Foundation (Grant No. OPP1017093). Additional support to International Vaccine Institute is provided by the governments of the Republic of Korea and Sweden. Ayan Dey was supported by National Research foundation of Korea (http://www.nrf.re.kr), grant number 2013K1AZA1058633.

### Competing interest statement

The authors declare no conflict of interest.

### Additional information
