# Supplementary material for: Comparison of IPV to tOPV week 39 boost of primary OPV vaccination in Indian infants: an open labelled randomized controlled trial
Source: Heliyon. 2017 Jan 9;3(1):e00223. doi: 10.1016/j.heliyon.2016.e00223 (PMC5289926; doi:10.1016/j.heliyon.2016.e00223)
Supplement: Supplementary Table 1 [file mmc1.docx]

**Table S1. Work plan for PROVIDE study**

| Clinic Visit # | | | | 0 | | | 1 | | 2 | | | 3 | | | 4 | 5 | | 6 | 7 | | 8 | 9 | | 10 | | 11 | | 12 |
| --- | --- | --- | --- | --- | --- | --- | --- | --- | --- | --- | --- | --- | --- | --- | --- | --- | --- | --- | --- | --- | --- | --- | --- | --- | --- | --- | --- | --- |
| Target Visit Schedule | | | | Birth | | | 6  wks | | 10  wks | | | 14  wks | | | 16  Wks | 17  wks | | 18  wks | 24  wks | | 39  wks | 40  wks | | 51  wks | | 52  wks | | 53-54  wks |
| EPI Vaccinations  (+)IPV  (-) IPV | | | | OPV,  BCG | | | DPT,  HepB,  OPV | | DPT,  HepB,  OPV  Rota#1 | | | DPT,  HepB,  OPV | | |  | Rota#2 | |  |  | | OPV | Measles | |  | | OPV | |  |
|  |  |  |  |  |  |  |  |  |  |  |  |  |  |  |  |  |  |  |  |  |  |  |  |  |  |  |  |  |
|  |  |  |  |  |  |  |  |  |  |  |  |  |  |  |  |  |  |  |  |  | IPV |  |  |  |  |  |  |  |
| Infant blood draws | | | |  | | | **2mL** | |  | | |  | | |  |  | | **2mL** |  | |  | **2mL** | |  | |  | | **4mL** |
| Breast milk IgA: rotavirus | | | |  | | | 5 mL | |  | | |  | | |  |  | |  |  | |  |  | |  | |  | |  |
| Maternal plasma neutralizing antibody: polio | | | |  | | | **5 mL** | |  | | |  | | |  |  | |  |  | |  |  | |  | |  | |  |
|  |  |  |  | | | **Poliovirus Vaccine Measures** | | | | | | | | | | | | | | | | | | | | | | |
| Antibody secreting cells/ ELISPOT | | | | |  | | X |  | | |  | | |  | | |  |  | |  |  | | X | |  | |  | X |
| Fecal excretion of vaccine virus* | | | | |  | |  |  | | | XXX | | |  | | |  |  | |  |  | |  | |  | | XXX |  |
| Specimen storage for Polio T-cells assay | | | | |  | |  |  | | |  | | |  | | |  |  | |  |  | |  | |  | |  | X |
| Plasma neutralizing antibody | | | | |  | | X |  | | |  | | |  | | |  | X | |  |  | | X | |  | |  | X |
|  |  |  |  | | | **Rotavirus Vaccine Measures** | | | | | | | | | | | | | | | | | | | | | | |
| Antibody secreting cells | | | | |  | | X |  | | |  | |  | | | |  | X | |  |  | |  | |  | |  |  |
| Plasma IgA | | | | |  | | X |  | | |  | |  | | | |  | X | |  |  | |  | |  | |  |  |
| Specimen storage for Rotavirus T-cells assay | | | | |  | |  |  | | |  | |  | | | |  |  | |  |  | |  | |  | |  | X |
|  |  |  |  | | | **Systemic Vaccine Measure** | | | | | | | | | | | | | | | | | | | | | | |
| Plasma IgG for Tetanus | | | | |  | |  |  | | |  | | |  | | |  | X | |  |  | |  | |  | |  |  |
|  |  |  |  | | | **Nutritional Measures** | | | | | | | | | | | | | | | | | | | | | | |
| Anthropometry (HAZ,WAZ) | | | | | X | | X | X | | X | | | | X | | | X | X | | X | X | | X | | X | | X | X |
| micronutrient assays  (RBP, Vit D, ferritin) | | | | |  | | X |  | |  | | | |  | | |  | X | |  |  | |  | |  | |  |  |
|  |  |  |  | | | **Tropical Enteropathy Measures** | | | | | | | | | | | | | | | | | | | | | | |
| Lactulose-mannitol ratio urine | | | | |  | |  |  | |  | | | X | | | |  |  | |  |  | | X | | X | |  |  |
| CRP, α-LPS/endoCAb | | | | |  | | X |  | |  | | |  | | | |  | X | |  |  | | X | |  | |  | X |
