# Supplementary material for: Comparison of IPV to tOPV week 39 boost of primary OPV vaccination in Indian infants: an open labelled randomized controlled trial
Source: Heliyon. 2017 Jan 9;3(1):e00223. doi: 10.1016/j.heliyon.2016.e00223 (PMC5289926; doi:10.1016/j.heliyon.2016.e00223)
Supplement: Table S2 [file mmc2.docx]

**Table S2. Baseline characteristic of the PROVIDE study cohort**

| **Characteristics** | **IPV**  **(n=186)** | **tOPV**  **(n=186)** | **p-value** |
| --- | --- | --- | --- |
| Mean (SD) age (in days) at enrollment | 44.75 (1.89) | 44.81 (2.09) | 0.94 |
| Mean (SD) age (in years) of mother at enrollment | 23.55 (4.39) | 23.53 (4.00) | 0.67 |
| N (%) boys | 92 (49.5) | 97 (52.2) | 0.60 |
| N (%) taken supplementation during pregnancy | 185 (99.5) | 183 (98.4) | 0.62 |
| N (%) taken zinc supplementation: Zinc | 11 (6.0) | 10 (5.4) | 0.83 |
| N (%) taken iron supplementation: Iron | 185 (99.5) | 183 (98.4) | 0.62 |
| N (%) taken folic acid supplementation: Folic acid | 183 (98.4) | 179 (96.2) | 0.20 |
| N (%) taken calcium supplementation: Calcium | 172 (92.5) | 170 (91.4) | 0.70 |
| N (%) taken Vit A supplementation: Vit A | 4 (2.2) | 1 (0.5) | 0.37 |
| N (%) received three of more doses of TET | 184 (98.9) | 185 (99.5) | 1.00 |
| N (%) Breastfed since birth | 158 (84.9) | 152 (81.7) | 0.40 |
| N (%) wasting (WHZ <-2) infants | 15 (8.1%) | 14 (7.5%) | 0.85 |
| Mean (SD) mother’s weight at enrolment (in kg) | 50.31 (9.55) | 48.56 (9.77) | 0.05 |
| Mean (SD) mother’s height at enrolment (in cm) | 147.22 (5.47) | 146.04 (5.06) | 0.03 |
| Mean (SD) mother’s BMI at enrolment | 23.17 (3.96) | 22.68 (3.93) | 0.25 |
| N (%) underweight (BMI<18.5) mother | 14 (7.5) | 23 (12.4) | 0.19 |
| N (%) overweight (BMI>=25) mother | 51 (27.4) | 41 (22.0) | 0.19 |
| N (%) having primary education of mother | 147 (79.0) | 148 (79.6) | 0.90 |
| N (%) having primary education of father | 160 (86.0) | 150 (80.6) | 0.16 |
| N (%) washing both hands before feeding infants | 134 (72.0) | 137 (73.7) | 0.73 |
| N (%) washing both hands before eating | 169 (90.9) | 158 (84.9) | 0.08 |
| N (%) washing both hands after defecating | 166 (89.2) | 175 (94.1) | 0.09 |
| N (%) washing both hands before cleaning children’s bottle | 28 (15.1) | 33 (17.7) | 0.48 |
| N (%) washing both hands after cleaning children’s anus | 180 (96.8) | 178 (95.7) | 0.59 |
| N (%) owning of at least one luxury item (any one of telephone/mobile, working TV, and motorcycle) | 184 (98.9) | 183 (98.4) | 1.00 |
| Mean (SD) expenditure (in Indian Rupees)) | 6220 (3848.8) | 5685 (2400.8) | 0.57 |
| N (%) using safe water for drinking (Municipality supply/piped water, Tube well, Protected well) | 186 (100.0) | 186 (100.0) | -- |
| N (%) using safe sanitation (Septic tank or toilet, Water-sealed or slab latrine) | 178 (97.3) | 175 (96.2) | 0.57 |
| N (%) treating water before drinking (Water filter, Solar disinfection, Boil, Strain through cloth, Add bleach/Chlorine) | 21 (11.3) | 26 (14.0) | 0.44 |
| N (%) having open drain beside house | 77 (41.4) | 73 (39.2) | 0.67 |
