# Supplementary material for: Comparison of IPV to tOPV week 39 boost of primary OPV vaccination in Indian infants: an open labelled randomized controlled trial
Source: Heliyon. 2017 Jan 9;3(1):e00223. doi: 10.1016/j.heliyon.2016.e00223 (PMC5289926; doi:10.1016/j.heliyon.2016.e00223)
Supplement: Table S3 [file mmc3.docx]

**Table S3. Categories of Protocol Deviations**

| **Poliovirus Vaccination Protocol Deviation** | | **Count** |
| --- | --- | --- |
| Termination | terminated | 1 |
|  | Week 14 missed vaccine AND terminated | 1 |
|  | Week 10+14 missed vaccine AND terminated | 12 |
|  | Week 39 missed vacconeAND terminated | 12 |
|  | Week 52 missed vax AND terminated | 4 |
|  | **sub-total** | **30** |
| One or more missed vaccination visits | Week 39 missed vaaccine | 1 |
|  | Week 52 missed vaccine | 1 |
|  | **sub-total** | **2** |
| One or more vaccination visits outside window | Week 6+10+14 outside window | 1 |
|  | Week 10 outside window | 13 |
|  | Week 10+14 outside window | 16 |
|  | Week 14 outside window | 39 |
|  | Week 52 outside window | 7 |
|  | Week 39 outside window | 5 |
|  | **sub-total** | **81** |
| **Total** | | **113** |

Note: The week X outside window means that the infant visit at week X occurred outside protocol specified time window. The week X missed vaccination means that the infant didn’t receive week X vaccination. The allowed time window for the visits at age 6, 10, 14, 39, and 52 weeks were +7 days. For the fecal sampling series at age 52 weeks, the allowed stool collection window for day 4, 11, 18, and 25 was +1 day. For day 0, where the challenge tOPV dose was administered, the window was -1 day. The 259 infants included in PP analysis and the 340 infants included in RPD analysis.
