# Supplementary material for: Comparison of IPV to tOPV week 39 boost of primary OPV vaccination in Indian infants: an open labelled randomized controlled trial
Source: Heliyon. 2017 Jan 9;3(1):e00223. doi: 10.1016/j.heliyon.2016.e00223 (PMC5289926; doi:10.1016/j.heliyon.2016.e00223)
Supplement: Table S4 [file mmc4.docx]

**Table S4. Distribution of all Serious Adverse Events by trial arm**

| Outcome | Event Term | Event Severity | IPV arm | tOPV arm | P-value |
| --- | --- | --- | --- | --- | --- |
| Death | Unknown | Severe | 0 | 1 | - |
|  | Total number of infants | | 0 (0%) | 1 (0.5%) | 1.00 |
| Hospitalization | Sepsis | Severe | 1 | 0 | - |
|  | Respiratory Illness | Mild | 3 | 0 | - |
|  |  | Moderate | 1 | 2 | - |
|  |  | Severe | 2 | 8 | - |
|  | Respiratory distress | Mild | 0 | 1 | - |
|  |  | Severe | 0 | 2 | - |
|  | Diarrheal Illness | Mild | 1 | 1 | - |
|  |  | Moderate | 1 | 0 | - |
|  |  | Severe | 1 | 4 | - |
|  | Febrile Illness | Mild | 1 | 0 | - |
|  | Total number of Events | | 11 | 18 | - |
|  | Total number of infants | | 11 (6%) | 8 (4%) | 0.48 |
| Total | | | 11 (6%) | 9 (5%) | 0.65 |

Note: The events were after completion of the poliovirus trial protocol period ending on the day 25 poliovirus excretion fecal sample visit at age 52 weeks.
