# Supplementary material for: Comparison of IPV to tOPV week 39 boost of primary OPV vaccination in Indian infants: an open labelled randomized controlled trial
Source: Heliyon. 2017 Jan 9;3(1):e00223. doi: 10.1016/j.heliyon.2016.e00223 (PMC5289926; doi:10.1016/j.heliyon.2016.e00223)
Supplement: Table S5 [file mmc5.docx]

**Table S5. Distribution of cases received OPV doses during Pulse Polio Immunization program (before week 40 visit) among subjects included in ITT analysis**

| No. of pulse OPV doses received | IPV (n=186) | | tOPV (n=186) | | p-value |
| --- | --- | --- | --- | --- | --- |
|  | Cases/mean | %/Std | Cases/mean | %/Std |  |
| 0 | 5 | 3 | 3 | 2 | 0.80 |
| 1 | 7 | 4 | 8 | 4 |  |
| 2 | 11 | 6 | 16 | 9 |  |
| 3 | 62 | 33 | 65 | 35 |  |
| 4 | 82 | 44 | 80 | 43 |  |
| 5 | 19 | 10 | 14 | 8 |  |
| Mean number of pulse OPV | 3.43 | 1.06 | 3.46 | 1.01 | 0.51 |
