# Supplementary material for: Comparison of IPV to tOPV week 39 boost of primary OPV vaccination in Indian infants: an open labelled randomized controlled trial
Source: Heliyon. 2017 Jan 9;3(1):e00223. doi: 10.1016/j.heliyon.2016.e00223 (PMC5289926; doi:10.1016/j.heliyon.2016.e00223)
Supplement: Table S6 [file mmc6.docx]

**Table S6. Presence of poliovirus Sabin type in fecal samples (secondary analysis)**

|  | No. shed/total (%) | | Risk Difference  (95% CI) | Relative Risk  (95% CI) | P value |
| --- | --- | --- | --- | --- | --- |
|  | IPV arm | tOPV arm |  |  |  |
| ***Cell culture*** | | | | | |
| Received protocol dose | 52/173 (30.1%) | 51/167 (30.5%) | -0.5%  ( -9, 7.7) | 0.98  (0.71, 1.36) | 0.92 |
| Per protocol | 46/133 (34.6%) | 42/126 (33.3%) | 1.3%  ( -8, 11) | 1.04  (0.74, 1.46) | 0.83 |
| ***Cell culture: infants with complete shedding data*** | | | | | |
| Received protocol dose | 49/152 (32.2%) | 45/147 (30.6%) | 1.6%  ( -7, 10) | 1.05  (0.75, 1.47) | 0.76 |
| Per protocol | 43/123 (35%) | 37/111 (33.3%) | 1.6%  ( -9, 12) | 1.05  (0.73, 1.50) | 0.79 |
| ***Fecal RT-qPCR:*** | | | | | |
| Received protocol dose | 52/173 (30.1%) | 51/167 (30.5%) | -0.5%  ( -9, 7.7) | 0.98  (0.71, 1.36) | 0.92 |
| Per protocol | 46/133 (34.6%) | 42/126 (33.3%) | 1.3%  ( -8, 11) | 1.04  (0.74, 1.46) | 0.83 |
